# Multi-Task Linear Programming Discriminant Analysis for the Identification of Progressive MCI Individuals

**DOI:** 10.1371/journal.pone.0096458

**Published:** 2014-05-12

**Authors:** Guan Yu, Yufeng Liu, Kim-Han Thung, Dinggang Shen

**Affiliations:** 1 Department of Statistics and Operations Research, The University of North Carolina at Chapel Hill, Chapel Hill, North Carolina, United States of America; 2 Department of Biostatistics, The University of North Carolina at Chapel Hill, Chapel Hill, North Carolina, United States of America; 3 Carolina Center for Genome Sciences, The University of North Carolina at Chapel Hill, Chapel Hill, North Carolina, United States of America; 4 Department of Radiology and Biomedical Research Imaging Center, The University of North Carolina at Chapel Hill, North Carolina, United States of America; 5 Department of Brain and Cognitive Engineering, Korea University, Seoul, Korea; Beijing Normal University, China

## Abstract

Accurately identifying mild cognitive impairment (MCI) individuals who will progress to Alzheimer's disease (AD) is very important for making early interventions. Many classification methods focus on integrating multiple imaging modalities such as magnetic resonance imaging (MRI) and fluorodeoxyglucose positron emission tomography (FDG-PET). However, the main challenge for MCI classification using multiple imaging modalities is the existence of a lot of missing data in many subjects. For example, in the Alzheimer's Disease Neuroimaging Initiative (ADNI) study, almost half of the subjects do not have PET images. In this paper, we propose a new and flexible binary classification method, namely Multi-task Linear Programming Discriminant (MLPD) analysis, for the incomplete multi-source feature learning. Specifically, we decompose the classification problem into different classification tasks, i.e., one for each combination of available data sources. To solve all different classification tasks jointly, our proposed MLPD method links them together by constraining them to achieve the similar estimated mean difference between the two classes (under classification) for those shared features. Compared with the state-of-the-art incomplete Multi-Source Feature (iMSF) learning method, instead of constraining different classification tasks to choose a common feature subset for those shared features, MLPD can flexibly and adaptively choose different feature subsets for different classification tasks. Furthermore, our proposed MLPD method can be efficiently implemented by linear programming. To validate our MLPD method, we perform experiments on the ADNI baseline dataset with the incomplete MRI and PET images from 167 progressive MCI (pMCI) subjects and 226 stable MCI (sMCI) subjects. We further compared our method with the iMSF method (using incomplete MRI and PET images) and also the single-task classification method (using only MRI or only subjects with both MRI and PET images). Experimental results show very promising performance of our proposed MLPD method.

## Introduction

Alzheimer's disease (AD) is one of the most common forms of dementia characterized by progressive cognitive and memory deficits. The increasing incidence of AD makes the disease a very important health issue and also huge financial burden for both patients and governments [1], [2]. It has been reported that 1 in every 85 persons in year 2050 will be likely affected by the disease [3]. The cost of care for AD patients by family members or health care professionals is more than 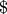
100 billion per year, and this number is expected to rise dramatically as the population ages during the next several decades [4]. As more and more treatments are being developed and evaluated, it is very important to develop diagnostic and prognostic biomarkers that can predict which individuals are relatively more likely to progress clinically. This kind of research is especially important for the individuals with Mild Cognitive Impairment (MCI), which is a prodromal stage of AD, since approximately 10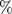 to 15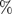 of individuals with MCI will progress to the probable AD [5], [6] although other MCI individuals remain stable. Hence, there is much interest in the research of early diagnosis of AD to identify those MCI individuals who will progress to clinical AD (progressive MCI) from those who remain stable (stable MCI). This study is very valuable for making early interventions in order to prevent the onset of AD or at least reduce the risk.

It is known that AD is related to the structural atrophy, pathological amyloid depositions, and metabolic alterations in the whole brain [7], [8]. At present, neuroimaging techniques are very promising and powerful tools for the diagnosis of AD or MCI. For example, structural magnetic resonance imaging (MRI) [9]–[12] can be used to delineate brain atrophy, functional MRI (fMRI) can be used to characterizes hemodynamic response related to neural activity [13], [14], and fluorodeoxyglucose positron emission tomography (FDG-PET) can be used to measure the metabolic patterns in the brain [15], [16]. In the past decade, many classification methods have been developed for early diagnosis of AD and MCI, including methods based on the structural MRI data only [6], [17]–[19] and methods based on both MRI and PET data [20]–[24]. Other studies about the diagnosis of AD and MCI can be found in [25]–[32]. Since different brain imaging modalities provide the complementary information for characterizing brain structures and functions, the classification methods using both MRI and PET data are shown to deliver much better performances.

One special challenge for using multi-modality data for disease diagnosis is related to the missing data, which is unavoidable, i.e., due to the high cost of measures (e.g., PET scans) or patients' dropout. In the Alzheimer's Disease Neuroimaging Initiative (ADNI) study, almost half of the subjects do not have PET images. Most existing classification methods using both MRI and PET are not applicable to this dataset due to missing data. To address this issue, the simplest method is to remove all subjects/samples with missing PET images. However, this approach will greatly reduce the sample size and ignore a lot of useful information in the samples with missing PET. Another approach is to use the missing data imputation method, e.g., those in [33]–[36]. Although these algorithms could be effective when the missing locations are random, they are less effective when a complete block of the data (such as PET) is missing (in almost half of the ADNI subjects). The state-of-the-art method for integrating multi-source incomplete data is the incomplete Multi-Source Feature (iMSF) learning method proposed by [37]. The iMSF method formulates the prediction problem as a multi-task learning problem by first decomposing the prediction problem into a set of tasks, one for each combination of data sources available, and then building the models for all tasks simultaneously. The important assumption in the iMSF method is that all models involving a specific source share the common set of features for that particular source. However, when different imaging modalities are highly correlated, this assumption could be too strong. In that case, for some data sources, it may be more reasonable to choose different feature subsets for different involved tasks.

In this paper, we propose a new and flexible binary classification method, namely Multi-task Linear Programming Discriminant (MLPD) analysis. The proposed MLPD method can be viewed as a generalization of the Linear Programming Discriminant (LPD) rule [38] for single-task classification. As the iMSF method, MLPD method also formulates the learning of multi-source incomplete data as a multi-task learning problem. Specifically, to jointly solve all those different tasks together, our proposed MLPD method constrains them to achieve the similar estimated mean difference between two classes (under classification) for those shared features. For a specific data source, instead of constraining all involved tasks to share the common set of features, MLPD could flexibly and adaptively select different feature subsets for different tasks. Furthermore, MLPD can be efficiently implemented by linear programming. As an illustrative application, we will combine the incomplete MRI and PET images to discriminate between progressive MCI and stable MCI. Our experimental results show very competitive performance of our proposed MLPD method.

The rest of this paper is organized as follows. In the Materials section, we describe the data set and the image preprocessing procedure. In the Method section, we review some important learning methods and then introduce our proposed MLPD method in detail. In the Experimental Result section, we compare our method with other state-of-the-art methods. In the Discussion section, we further discuss different classification methods, and also the effect of parameter and the methodological limitations in our proposed method. Finally, we conclude this paper in the Conclusion section.

## Materials

Data used in the preparation of this paper were obtained from the Alzheimer's Disease Neuroimaging Initiative (ADNI) database (http://adni.loni.ucla.edu/). As a 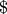
60 million, 5-year public-private partnership, the ADNI was launched in 2003 by the National Institute on Aging (NIA), the National Institute of Biomedical Imaging and Bioengineering (NIBIB), the Food and Drug Administration (FDA), private pharmaceutical companies and non-profit organizations. The primary goal of ADNI has been to test whether serial magnetic resonance imaging (MRI), positron emission tomography (PET), other biological markers, and clinical and neuropsychological assessments can be combined to measure the progression of mild cognitive impairment (MCI) and early Alzheimer's disease (AD). Determination of sensitive and specific markers of very early AD progression is very important and useful for the researchers and clinicians to develop new treatments and monitor their effectiveness as well as lessen the time and cost of clinical trials.

The Principal Investigator of ADNI is Michael W.Weiner who is a MD at VA Medical Center and University of California-San Francisco. ADNI is the results of efforts of many co-investigators from a broad range of academic institutions and private corporations. Subjects in that research have been recruited from over 50 sites across the U.S. and Canada. The initial goal of ADNI was to recruit 800 adults with age between 55 and 90. Approximately, 200 cognitively normal older individuals and 400 MCI individuals were followed for 3 years and 200 individuals with early AD were followed for 2 years (see www.adni-info.org for up-to-date information).

According to some criteria such as Mini-Mental State Examination (MMSE) scores, the subjects in ADNI can be divided into three categories: healthy subjects, MCI subjects and AD. The detailed description for each category can be found in [23]. For MCI, some subjects had converted to AD within 18 months, while some other MCI subjects were stable over time [5], [6]. Based on whether the MCI subjects would convert to AD within 18 months, the MCI subjects are divided into two classes: progressive MCI (pMCI) and stable MCI (sMCI). In this paper, only MCI subjects with corresponding MRI and/or PET baseline data are used. This yields a total of 393 MCI subjects, including 167 pMCI and 226 sMCI. However, some MCI subjects have both MRI and PET baseline data, while some MCI subjects have only MRI baseline data. All subject information is summarized in Table 1.

**Table 1:** Summary of subject information.

Class label	Category	Sample size	Age	Female/Male	Education	MMSE
pMCI	MRI+PET	76	75.4 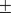 6.5	28/48	15.9 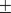 2.6	26.7 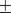 1.7
	MRI	91	74.4 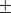 7.2	37/54	15.5 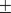 3.0	26.5 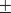 1.7
sMCI	MRI+PET	126	74.9 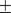 7.4	38/88	15.6 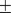 3.0	27.4 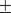 1.7
	MRI	100	75.0 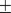 8.0	37/63	15.5 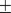 3.4	27.1 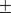 1.9

(For the second column, MRI+PET represents the group of subjects with both MRI and PET features, and MRI represents the group of subjects with only MRI features. The last column shows the Mini-Mental State Examination (MMSE) score.).

### MRI and PET

All structural MR scans were acquired from 1.5T scanners. We downloaded the raw Digital Imaging and Communications in Medicine (DICOM) MRI scans from the public ADNI web site (www.loni.ucla.edu/ADNI). All the MR scans were reviewed for quality and automatically corrected for spatial distortion caused by gradient nonlinearity and 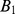 field inhomogeneity. The baseline PET data are also downloaded from the ADNI web site. PET images were acquired 30–60 min post-injection, averaged, spatially aligned, interpolated to a standard voxel size, intensity normalized and smoothed to a common resolution of 8-mm full width at half maximum.

### Image Processing

The image pre-processing is performed for all MRI and PET images, with the detailed process described below:

1. Use MIPAV software (http://mipav.cit.nih.gov/clickwrap.php) to perform anterior commissure (AC) - posterior commissure (PC) correction on all images and re-sample the images to the size of 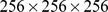;
2. Use the N3 algorithm [39] to correct the intensity inhomogeneity;
3. Perform skull-stripping using the method proposed in [40] on all the MRI images and then manually review each skull-stripping result to ensure the clean skull and dura removal;
4. Carry out the intensity inhomogeneity correction [39] and remove the cerebellum based on registration with atlas;
5. Use FAST in FSL [41] to segment each skull-stripped brain into three tissues: Grey Matter (GM), White Matter (WM) and Cerebrospinal Fluid (CSF);
6. Use HAMMER [42] to do the registration and further get the ROI-labeled image based on the Jacob template [43] with 93 manual ROIs;
7. From each of the 93 labeled ROIs in the MRI, compute its GM tissue volume as a feature;
8. Align the PET image to its respective MR image of the same subject using affine registration, and then compute the average intensity of each ROI in the PET image as a feature.

After the above image preprocessing, for each subject, we obtain 93 features from MRI and/or other 93 features from PET image (depending on whether PET image is available for this subject).

## Ethics Statement

The individuals in this manuscript have given written informed consent at the time of enrollment for imaging and completed questionnaires approved by each participating sites. The authors have obtained approval from the ADNI Data Sharing and Publications Committee to use the data. The authors confirm that the data was analyzed anonymously.

## Methods

Suppose our data were collected from 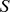 data sources, e.g., 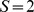 with MRI and PET in this study. Note that, in this study, the complete block of the data (such as PET) was missing in many subjects, thus there exists multiple possible combinations of data sources available. In this way, it is natural to build multiple classifiers for multiple combinations, with one classifier for one possible combination. Rather than learning each classifier separately, which could limit the overall performance, we propose to learn multiple classifiers jointly by using the whole data set. This can be formulated as a multi-task learning problem, where one task represents learning one classifier. Suppose that our dataset leads to totally 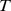 different combinations for the available data sources. Then we have 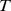 tasks in our formulated multi-task learning framework, where subjects in the same task have the same number of features. For the *i*th task, we denote the number of features as 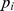 and the training subjects as 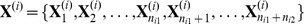, where 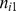 (or 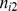) is the number of subjects in the first class (or second class) and 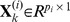 represents features of the *k*th subject in the *i*th task. Furthermore, we denote 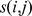 as the set of the shared features indices between task 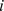 and task 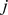.

In the following sections, we first review the classic linear discriminant analysis (LDA) and also the recent linear programming discriminant (LPD) rule for single-task learning. Then, we introduce our proposed MLPD method for multi-task learning in detail.

### Linear Discriminant Analysis and Linear Programming Discriminant Rule

If all the features are observed and the number of subjects is much larger than the number of features, linear discriminant analysis (LDA), which uses a linear combination of features to separate two classes of subjects, has been shown to perform well in machine learning. Without loss of generality, we use data in task 1 (described above), 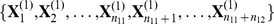, to illustrate how to construct a linear classifier based on LDA.

LDA assumes that 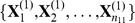 and 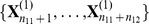 are the independently and identically distributed random samples from 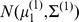 and 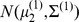 respectively. If 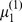, 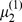 and 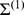 are known, the LDA method classifies a new subject 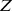 to class 1 if and only if
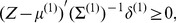
(1) where 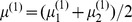 and 
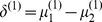
.

As shown in (1), LDA requires the estimation of the inverse covariance matrix. In the low dimensional setting, the inverse sample covariance matrix can be a good estimation. However, in the high dimensional setting, the sample covariance is singular and its inverse is not well defined. In that case, certain structural assumptions on 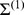 (or 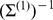) and 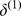 are needed to ensure that the estimations are consistent [38]. The most commonly used structural assumption is the sparsity assumption. Under the assumption that 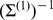 and 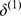 are sparse, 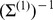 and 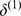 can be estimated separately and then plugged into the LDA [44], [45].

In the high dimensional setting, a recent important extension of LDA method is introduced in [38]. As shown in (1), it can be observed that the LDA method depends on 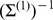 and 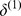 only through their product 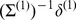. In the proposed LPD rule [38], instead of estimating 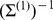 and 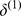 separately, they suggested to estimate the product 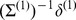 directly through a constrained 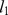 minimization method. The LPD rule performs well when 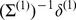 is approximately sparse, which is a weaker and more flexible assumption than requiring both 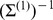 and 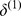 to be sparse. Theoretically, the LPD rule can get the asymptotically optimal misclassification rate under some conditions. For computation, this method can be implemented efficiently by linear programming.

Considering task 1, we can construct the single-task LPD (SLPD) rule as follows:

1. Compute

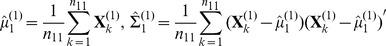


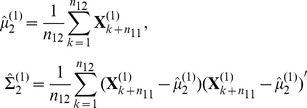


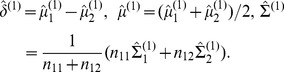

2. Compute

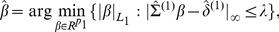
(2)where 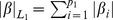, 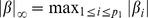 and 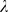 is a tuning parameter chosen by cross validation.

3. For a testing subject 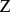, classify 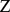 to the first class if and only if
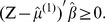

The optimization problem in (2) is convex and can be recast as the following linear programming problem:
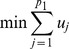


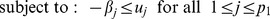


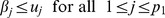


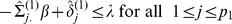


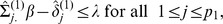
 where 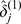 is the *j*th component of 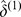 and 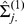 is the *j*th row of 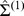.

### Multi-task Linear Programming Discriminant (MLPD) Analysis

As mentioned above, for the incomplete data collected from multiple data sources, we can decompose the respective classification problem into several different tasks. Since different tasks could share some common features, these tasks can be highly related. Instead of learning each task separately, it is very important and useful to learn all these tasks simultaneously. For the multi-task learning, the most important issue is how to link different tasks. In the existing multi-task learning methods, some methods assume that different tasks share parameters or prior distributions of the hyper-parameters [46], while other methods assume that different tasks share a common underlying representation [37], [47]. In this paper, since different tasks correspond to different combinations of data sources, we propose to link different tasks by requiring them to achieve the similar estimated mean difference between two classes (under classification) for those shared features.

In order to use all the available incomplete multi-source data, we generalize the LPD rule as follows:

1. For 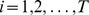, compute

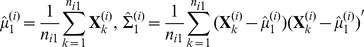


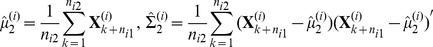


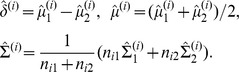

2. Estimate 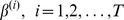 as follows:

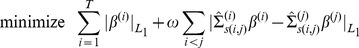
(3)

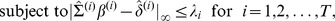
where 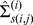 is the sub-matrix of 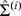 with row indices in the common feature set 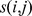.

3. Denote 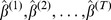 as the solutions for the optimization problem (3). For testing subject 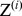 which has the same features as the training subjects in task 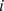, classify 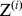 to the first class if and only if

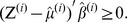



Note that, if the parameter 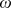 in (3) is set to be zero, the LPD rule for multiple tasks is the same as the LPD rule for the single task. The term 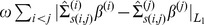 in (3) links different tasks together. In fact, 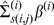 estimates 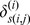, which is the expected mean difference of features 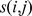 between class 1 and class 2 in the *i*th task. Since the features in the set 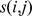 are shared by task 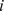 and task 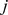, the difference between the estimate of 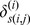 and the estimate of 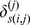 should be small. The parameter 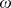 is used to control their similarity. Similar to the optimization problem in (2), problem in (3) is also convex and thus can be formulated as a linear programming problem.

### MLPD for the incomplete MRI and PET images

**Figure 1:**
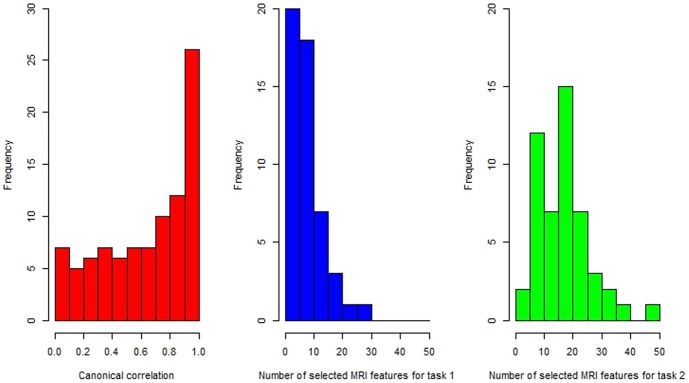
**Left**: Histogram of the canonical correlations between MRI features and PET features; **Middle**: Histogram of the number of selected MRI features for task 1 based on 50 times simulation. Each time we chose 76 pMCI subjects and 76 sMCI subjects randomly in task 1; **Right**: Histogram of the number of selected MRI features for task 2 based on 50 times simulation. Each time we chose 76 pMCI subjects and 76 sMCI subjects randomly in task 2. For the middle and right plots, the LASSO method is used for feature selection and 10-fold cross validation is used to choose the optimal number of features.

For imaging modalities MRI and PET, even after feature extraction, there are still many irrelevant features. Thus, feature selection is a very important step for removing those irrelevant features before learning a good classifier. The state-of-the-art iMSF method constrains all models involving a specific data source to select a common set of features for that particular data source. This constraint could be too rigorous, especially when there exists a strong correlation between different imaging modalities such as MRI and PET used in this paper. Specifically, all 393 subjects have MRI features, while only 202 subjects have both MRI and PET features. Then, we can decompose this classification problem into two tasks: task 1 for subjects with both MRI and PET features, and task 2 for subjects with only MRI features. The canonical correlations between MRI features and PET features using the data in task 1 are shown in Figure 1. The canonical correlation histogram indicates strong correlation between MRI features and PET features. Since PET features can explain some information of MRI features, instead of choosing the same MRI features as task 1 (with both MRI and PET), it may be more reasonable to select more MRI features for task 2 (with only MRI) in order to achieve reasonable predictive performance. The histograms of the number of selected features for each task by the LASSO method [48] as shown in Figure 1 further demonstrate this point.

For our case of using two data sources (MRI and PET), each subject in task 1 has 186 features, while each subject in task 2 has 93 features. In order to save the computational time, we use the two-sample t-test method to remove some obvious noisy features before using our proposed MLPD method. Specifically, for each MRI feature and PET feature, we compute the p-value of the two-sample t-test only using the training subjects in task 1 and denote the respective p-values as 
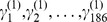
. Furthermore, for each MRI feature, we compute the p-value of the two-sample t-test only using the training subjects in task 2 and denote the respective p-values as 
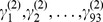
. For task 1, we remove feature 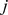 if 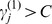. For task 2, in order to keep more MRI features, we remove feature 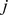 if 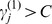 and 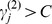. Here, 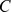 is a predefined threshold parameter.

After removing those noisy features, we reorder the features so that common MRI features of task 1 and task 2 are the first 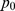 features. Similar to the optimization problem in (2), our proposed MLPD method for this two-task learning problem can be recast as the following linear program:
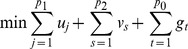


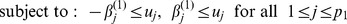


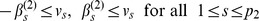


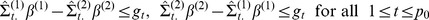


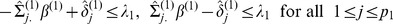


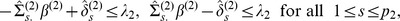
where 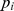 (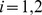) is the number of features in task 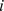, 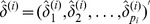 and 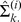 is the *k*th row of 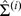.

## Results

In this section, we perform various experimental studies to demonstrate the effectiveness of our proposed MLPD method. As mentioned, two imaging modalities (MRI and PET) are used in our experiment. The data set contains 167 pMCI subjects and 226 sMCI subjects. Among all these 393 subjects, 76 pMCI and 126 sMCI subjects have both MRI and PET features, while 91 pMCI and 100 sMCI subjects have only MRI features. Our experiments include two separate parts. In the first part, we compare MLPD method with the state-of-the-art iMSF method for the incomplete multi-source feature learning. In the second part, in order to show the advantage of using the whole incomplete data set, we compare MLPD with SLPD (Single-task Linear Programming Discriminant) when considering subjects with only MRI features or subjects with both MRI and PET features.

### Experimental Setup

We use 2-, 5- and 10-fold cross validation (CV) strategies respectively to evaluate the classification accuracy. Specifically, when 10-fold CV is used, 76 pMCI (task 1), 126 sMCI (task 1), 91 pMCI (task 2), and 100 sMCI (task 2) subjects are equally partitioned into 10 subsets, respectively. Each time the subjects within one subset are selected as the testing subjects, while the subjects in the other 9 subsets are used as the training subjects. For both MLPD and SLPD methods, the parameter 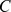 used in the previous two-sample t-test based feature selection step is fixed as 0.01. The effect of the parameter 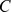 will also be discussed in the Discussion section. For the SLPD method, 20 equally spaced values of 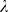 in logarithmic scale between 0.01 and 1 are considered, and then the inner 5-fold CV on the training data is used to choose the optimal 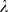. Similarly, for MLPD, we also use the same 5-fold CV strategy on the training data to select its respective parameters such as 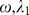, and 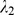. Specifically, for 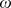, 20 equally spaced values in logarithmic scale between 0.01 and 10 are searched. For 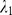, we use the same sequence as 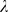. For 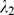, we set 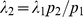 where 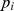 is the total number of features in task 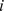.

In the experiment, we treat pMCI as positive class and sMCI as negative class. The performances of different methods are evaluated by accuracy, sensitivity, and specificity, as defined below:
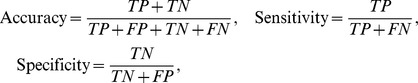
where 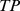, 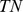, 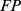 and 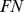 denote numbers of true positives, true negatives, false positives and false negatives, respectively.

Here, the sensitivity measures the proportion of true pMCI that are correctly identified, and the specificity measures the proportion of sMCI that are correctly identified. The accuracy measures the overall correct classification rate for the whole data set. The whole process is repeated 30 times and the average performance is reported.

### Comparison of MLPD with iMSF using incomplete MRI and PET

In our first experiment, we apply both MLPD and iMSF methods for the incomplete multi-source dataset including MRI and PET. The experiment setup for MLPD has been described above. For iMSF method [37], the multi-task feature learning framework is given by
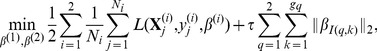
(4)where 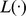 is the loss function, 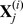 is the feature vector of the *j*th subject in the *i*th task and 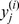 is the corresponding label value. Here 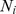 is the number of subjects in the *i*th task and 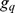 is the number of features from the *q*th data source. Furthermore, 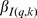 denotes all the model parameters corresponding to the *k*th feature in the *q*th data source.

In our experiment, we consider two types of loss functions for iMSF:

- Quadratic loss function: 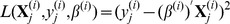;
- Logistic loss function: 
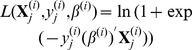
.

For each loss function, we again use 2-, 5- and 10-fold CV to evaluate the classification accuracy of iMSF. For parameter 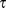, 10 equally spaced values in the interval [0.005, 0.4] are considered, and the optimal 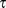 is determined with another inner 5-fold CV on the training data. For each of two loss functions (given above), we repeat the iMSF method 30 times and the average performance is reported.

Table 2 shows the evaluation results of MLPD and iMSF when all available data are used. It can be observed that our proposed MLPD method has better performance than iMSF. For cases using 5-fold CV and 10-fold CV, the classification accuracy of MLPD is around 2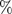 better than the iMSF method. Furthermore, the MLPD acquires much higher sensitivity. In this experiment, higher sensitivity means higher classification accuracy for the progressive MCI patients. It is worth noting that, in practice, the cost of misclassifying progressive MCI patients is usually much higher than that of misclassifying stable MCI patients. Thus, the high sensitivity characteristic of our proposed MLPD method is very useful for the identification of progressive MCI patients.

**Table 2:** Classification performance of MLPD and iMSF using incomplete MRI and PET.

pMCI(+1)/sMCI(−1)	k-fold CV	iMSFQ	iMSFL	MLPD
Accuracy	2	0.6433 (0.0030)	0.6420 (0.0035)	**0.6569** (0.0034)
	5	0.6467 (0.0023)	0.6482 (0.0031)	**0.6697** (0.0022)
	10	0.6581 (0.0022)	0.6578 (0.0030)	**0.6719** (0.0025)
Sensitivity	2	0.4913 (0.0072)	0.4965 (0.0061)	**0.6431** (0.0065)
	5	0.4853 (0.0063)	0.5069 (0.0054)	**0.6734** (0.0038)
	10	0.4906 (0.0040)	0.5223 (0.0038)	**0.6780** (0.0048)
Specificity	2	**0.7556** (0.0048)	0.7496 (0.0064)	0.6671 (0.0044)
	5	**0.7659** (0.0043)	0.7527 (0.0048)	0.6671 (0.0022)
	10	**0.7823** (0.0035)	0.7582 (0.0044)	0.6675 (0.0026)

(For this experiment, we used all the available data from 393 subjects in total. iMSFQ and iMSFL indicate the iMSF method using quadratic loss function and logistic loss function respectively. The best value for each performance measure is highlighted in bold. The value in the parenthesis is the standard deviation.).

In order to show the advantage of our proposed MLPD method, we also extract the classification results for each task when all the available data are used. Table 3 shows the classification performance on task 2. The results indicate that our proposed MLPD method has much higher classification accuracy than the iMSF methods. Specifically, for 5-fold CV, the classification accuracy of our method is around 4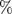 higher than the classification accuracy of the iMSF methods. This advantage of our proposed method is likely due to the flexible feature selection strategy used. For this data set, our proposed MLPD method chooses more MRI features for task 2, while iMSF methods (using two different loss functions) choose the same MRI features for each task. Due to limited space, we don't show the detailed evaluation results for task 1 here. For task 1, compared with the iMSF methods, our proposed MLPD method acquires similar classification accuracy while much higher sensitivity.

**Table 3:** Comparison of the classification performance of MLPD and iMSF on task 2.

pMCI(+1)/sMCI(−1)	k-fold CV	iMSFQ	iMSFL	MLPD
Accuracy	2	0.6363 (0.0047)	0.6386 (0.0055)	**0.6623** (0.0052)
	5	0.6291 (0.0039)	0.6346 (0.0041)	**0.6701** (0.0038)
	10	0.6321 (0.0038)	0.6380 (0.0043)	**0.6641** (0.0036)
Sensitivity	2	0.6026 (0.0101)	0.6052 (0.0092)	**0.6539** (0.0089)
	5	0.5990 (0.0074)	0.6104 (0.0060)	**0.6750** (0.0048)
	10	0.5996 (0.0057)	0.6146 (0.0067)	**0.6690** (0.0059)
Specificity	2	0.6670 (0.0062)	**0.6960** (0.0083)	0.6700 (0.0065)
	5	0.6563 (0.0049)	0.6567 (0.0055)	**0.6657** (0.0052)
	10	**0.6617** (0.0052)	0.6590 (0.0054)	0.6597 (0.0045)

(For this experiment, we used all the available data from 393 subjects in total. The classification results for the subjects in task 2 are reported. iMSFQ and iMSFL indicate the iMSF method using quadratic loss function and logistic loss function respectively. The best value for each performance measure is highlighted in bold. The value in the parenthesis is the standard deviation.).

### Comparison of MLPD with SLPD

In this section, we compare MLPD method with SLPD method in order to show the advantage of using the whole incomplete data set for diagnosis. The SLPD method is used in two cases. In the first case, we discard the PET features and then use SLPD method for all subjects. In the second case, we discard the subjects in task 2 and use SLPD method only for the subjects in task 1. Table 4 shows the comparison results for the MLPD method using all available data and the SLPD method only using MRI features. The results indicate that using both MRI and PET features improves the classification performance. Specifically, for 10-fold CV, the classification accuracy of MLPD is around 3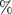 better than SLPD. Due to limited space, the detailed classification performance for only task 1 or only task 2 is not provided. But, briefly, when the additional PET features are used, the classification performance on task 1 is improved by around 5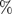 while the classification performance on task 2 is similar.

**Table 4:** Classification performance of MLPD and SLPD.

pMCI(+1)/sMCI(−1)	k-fold CV	SLPD	MLPD
Accuracy	2	0.6423 (0.0029)	**0.6569** (0.0034)
	5	0.6420 (0.0028)	**0.6697** (0.0022)
	10	0.6409 (0.0029)	**0.6719** (0.0025)
Sensitivity	2	**0.6533** (0.0036)	0.6431 (0.0065)
	5	0.6510 (0.0037)	**0.6734** (0.0038)
	10	0.6588 (0.0048)	**0.6780** (0.0048)
Specificity	2	0.6342 (0.0040)	**0.6671** (0.0044)
	5	0.6353 (0.0046)	**0.6671** (0.0022)
	10	0.6276 (0.0030)	**0.6675** (0.0026)

(For MLPD, all the available data are used. For SLPD, only the MRI features are used. The best value for each performance measure is highlighted in bold. The value in the parenthesis is the standard deviation.).

Table 5 shows the comparison results for the MLPD method using all available data and the SLPD method only using subjects in task 1. Note that, for the MLPD method, we extracted only the classification results for the subjects in task 1 and reported the performance, thus the comparison of two methods can be made on the same set of subjects. The comparison results in Table 5 indicate that subjects in task 2 could help build a better classifier for task 1 using our proposed MLPD method. Specifically, when using the subjects with missing PET features, the classification accuracy on the subjects with both MRI and PET features by our method can be improved by around 2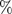.

**Table 5:** Comparison of the classification performance of MLPD and SLPD on task 1.

pMCI(+1)/sMCI(−1)	k-fold CV	SLPD	MLPD
Accuracy	2	0.6394 (0.0067)	**0.6518** (0.0045)
	5	0.6538 (0.0047)	**0.6694** (0.0036)
	10	0.6588 (0.0031)	**0.6793** (0.0033)
Sensitivity	2	0.6096 (0.0137)	**0.6303** (0.0089)
	5	0.6608 (0.0063)	**0.6715** (0.0058)
	10	0.6551 (0.0064)	**0.6884** (0.0058)
Specificity	2	0.6574 (0.0073)	**0.6648** (0.0068)
	5	0.6497 (0.0053)	**0.6682** (0.0042)
	10	0.6611 (0.0036)	**0.6736** (0.0038)

(For MLPD, all the available data is used, but the classification results for the subjects in task 1 is reported. For SLPD, only use the subjects in task 1. The best value for each performance measure is highlighted in bold. The value in the parenthesis is the standard deviation.).

In summary, all these experimental results show that, by making use of all available information, our proposed MLPD method can improve the overall classification performance.

## Discussion

In this section, we will further discuss the comparison of different classification methods, the effect of parameter 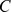 for the two-sample t-test based feature selection procedure, and the limitation of our MLPD method.

### Comparison between MLPD and other state-of-the-art methods

Our proposed MLPD method for the incomplete multi-source feature learning is able to flexibly and adaptively select features for different tasks, besides trying to solve them jointly. Compared with the methods that simply discard subjects with missing data or features which are not available for all subjects, our proposed MLPD method makes use of all available data and thus could achieve better classification performance. Specifically, MLPD formulates the original classification problem into a multi-task learning problem, with one task representing the learning of one classifier for one combination of available data sources. Compared with the state-of-the-art iMSF method, instead of selecting the same feature subset from the shared features for all involved tasks, MLPD could choose different “best” feature subsets for different tasks adaptively. This adaptive feature selection strategy is very important for the task with fewer available data sources, especially when these data sources are highly correlated with the other unavailable data sources. Note that, in order to use the whole available data, some data imputation algorithms such as [33]–[36] can be also used. However, these methods may be not effective for the data with large blocks of missing data, and in some cases, could be worse than the method of simply discarding the subjects with missing data.

### Effect of parameter 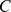


**Figure 2:**
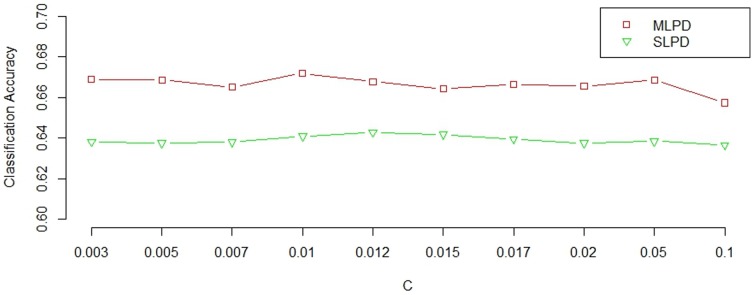
Classification accuracy of MLPD and SLPD with respect to different predefined threshold 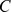.

The two-sample t-test is used to remove some obvious noisy features. Features with their p-values smaller than a predefined threshold parameter 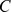 are preselected for our MLPD method. Larger value of 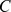 will preselect more features. In order to study the effect of the threshold parameter 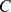, based on 10-fold cross validation, we try 10 different values for 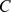, ranging from 0.003 to 0.1. For our MLPD method, when 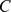 changes from 0.003 to 0.1, the average number of preselected features for task 1 changes from 12 to 60, while the average number of features preselected for task 2 changes from 22 to 50. Although the number of features preselected changes a lot, the classification accuracy of our MLPD method is always around 0.66. On the other hand, for the SLPD method, the average number of preselected features increases from 26 to 52 when changing parameter 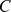 from 0.003 to 0.1, but its classification accuracy is always around 0.64. Figure 2 shows the classification accuracies of both MLPD and SLPD methods using different choices of 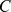. It can be observed that both MLPD and SLPD methods are relatively robust with respect to the number of features preselected. Furthermore, our proposed MLPDR method using incomplete MRI and PET data always acquires better classification accuracy than the SLPD method that uses only the MRI data.

### Limitations

Although our proposed MLPD method performs well in our experiments, there are still some methodological limitations. First, we used a simple two-sample t-test to preselect some features for each task, without considering the correlation between different features. It would be interesting to preselect more features for the task with fewer available data sources, using both label information and the correlation between different features. Second, our proposed MLPD method may require expensive computation if involving with many incomplete data sources. For example, if there are 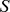 data sources and only one data source is complete, our MLPD method will decompose the classification problem into 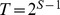 tasks. As shown in the optimization problem in (3), in addition to the variables assigned for each task, we also need to use many other variables representing the constraints for every two tasks. Thus, the linear programming (LP) algorithm will involve a large number of variables and accordingly more computational time. For that case, some decomposition methods [49], [50] should be used to decompose the complex LP problem (3) into a sequence of small LP problems.

## Conclusion

In summary, we have proposed a new incomplete data classification method for making full use of all available data, by formulating the original classification problem as a multi-task learning problem. Instead of requiring the different tasks involving a specific source to select a common set of features for that particular source, our MLPD method uses a more flexible feature selection strategy to allow for selection of different feature subsets for different tasks. Furthermore, it is very straightforward to formulate the optimization problem of MLPD as a linear programming problem, which can be efficiently solved by many software packages. Besides, we have also compared our method with several state-of-the-art methods, showing better classification performances.
